# A Case of Cholangitis Glandularis Proliferans and Cholangiocarcinoma of the Common Bile Duct

**DOI:** 10.1155/1993/85926

**Published:** 1993

**Authors:** A. J. Richardson, J. M. Grierson, N. Tait, S. J. Williams, J. M. Little

**Affiliations:** Department of Surgery Westmead Hospital Westmead N.S.W. 2145 Australia

## Abstract

A case of Cholangitis Glandularis Proliferans (CAGP) in association with a cholangiocarcinoma of the common bile duct as described. This is the eighth case of CAGP described and the second association with cholangiocarcinoma.


*HPB Surgery*, 1993, Vol. 6, pp. 205–209
Reprints available directly from the publisher
Photocopying permitted by license only
© 1993 Harwood Academic Publishers GmbH
Printed in the United States of America

### CASE REPORT

### A CASE OF CHOLANGITIS GLANDULARIS PROLIFERANS AND CHOLANGIOCARCINOMA OF THE COMMON BILE DUCT

A.J. RICHARDSON, J.M. GRIERSON, N. TAIT, S.J. WILLIAMS and J.M. LITTLE

*Department of Surgery, Westmead Hospital, Westmead N.S.W. 2145 Australia*

*(Received 13 January 1992)*

A case of Cholangitis Glandularis Proliferans (CAGP) in association with a cholangiocarcinoma of the common bile duct as described. This is the eighth case of CAGP described and the second association with cholangiocarcinoma.

KEY WORDS: Bile duct, cholangitis glandularis proliferans, cholangiocarcinoma

### INTRODUCTION

Cholangitis glandularis proliferans is a variant of sclerosing cholangitis characterised by proliferation of bile duct intramural glandular elements with concommitant inflammatory but not desmoplastic changes. It is an extremely rare condition with only 7 cases having been reported. The case described in this report occurred in association with a cholangiocarcinoma of the mid common bile duct. This association of cholangitis glandularis proliferans with bile duct malignancy has, to our knowledge, only been reported once before.

### CASE REPORT

A 39 year old male presented with a 10 day history of increasing jaundice and dark urine. Liver function tests were grossly abnormal, showing a cholestatic pattern.

Address correspondence to: Dr A.J. Richardson, Department of Surgery, Westmead Hospital, Westmead N.S.W. 2145 Australia

Significant past history included ulcerative colitis diagnosed 20 years previously and requiring intermittent Salazopyrin treatment. He had received no recent follow up for his colitis which had remained clinically quiescent.

CAT scanning revealed a mass surrounding the distal common bile duct with marked retroperitoneal lymphadenopathy. Endoscopic retrograde cholangiopancreatography showed a normal pancreatogram and an irregular stricture in the mid common bile duct consistent with a cholangiocarcinoma (Figure 1). The remainder of the common bile duct was slightly irregular and the intrahepatic ducts were dilated but regular in outline. Biliary decompression was achieved by endoprosthesis insertion and one week later he underwent laparotomy for frozen section examination of the enlarged lymph nodes and exploration of the bile duct mass.

At operation the presence of a mass in the distal common bile duct and associated marked lymphadenopathy were confirmed. Multiple lymph node biopsies showed no evidence of metastatic tumour. A radical pancreaticoduodenectomy was therefore performed including removal of the gallbladder and distal stomach. There were no stones present in the gallbladder.

Post-operatively, the patient made a good recovery and was fit for discharge two and a half weeks later. He remains well, six months after surgery. Colonoscopy subsequently showed quiescent colitis with no histological evidence of dysplasia or tumour.

### *Histology*

The sections from the common bile duct and ampullary ductal region showed moderately well differentiated adenocarcinoma with no extension beyond the planes of resection. None of the lymph nodes examined contained metastatic tumour. The common bile duct sections showed marked thickening due to cholangitis glandularis proliferans (CAGP) with intense inflammation, some mucosal ulceration, striking mucosal hyperplasia and moderate to severe epithelial atypia (Figure 2). Histopathology of the gallbladder showed moderate muscle thickening but no definite inflammatory changes. In particular, there was no evidence of cholecystitis glandularis proliferans (CGP).

### DISCUSSION

Distinctive histological features of cholangitis glandularis proliferans (CAGP) are the absence of fibrosis and the striking proliferation of bile duct intramural glandular elements in addition to the inflammatory changes similar to those seen in cholecystitis glandularus proliferans (CGP). Although it is attractive to suggest that the two processes share a common aetiology, only one case has been described where the two co-existed.[1] However a possible link between another biliary proliferative lesion, adenomyomatosis of the gall bladder, and gall bladder cancer has recently been reported.[2]

The association between typical sclerosing cholangitis and the subsequent development of cholangiocarcinoma is clear cut. It may be that CAGP is a less aggressive form of sclerosing cholangitis with a diminished but still significant propensity to neoplastic change. Our case is the second report in which cholangiocarcinoma has occurred in association with this condition[1], giving an incidence in

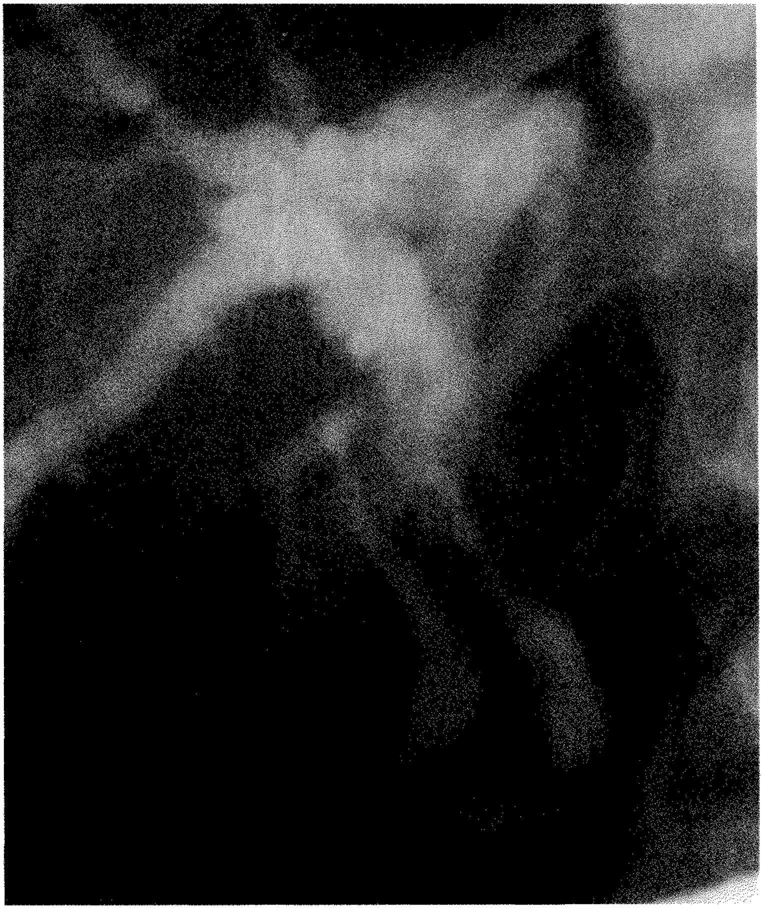

**Figure 1**

these reports of 25%. Whether the CAGP is truly a premalignant condition and if so, whether it is a variant of sclerosing cholangitis remains unanswered.

The underlying aetiology of CAGP remains obscure although it is conceivable that bile duct epithelium may be damaged or lost by inflammation or infection and that the surviving cells within the intramural tubular-alveolar gland proliferate in response to this injury[2,3]. The biliary epithelium is specifically adapted to the normal biliary lumenal milieu and changes in this milieu can induce adaptive alterations in the mucosal morphology and cell kinetics which may in turn play a role in the genesis of proliferative conditions such as CGP and CAGP. Florid adaptive metaplastic changes associated with intramural inflammation have been clearly demonstrated in bile duct epithelium adjacent to choledochoduodenostomies[5]. If mechanisms such as these are involved in the development of CAGP, it seems extraordinary that the condition is not seen more frequently.

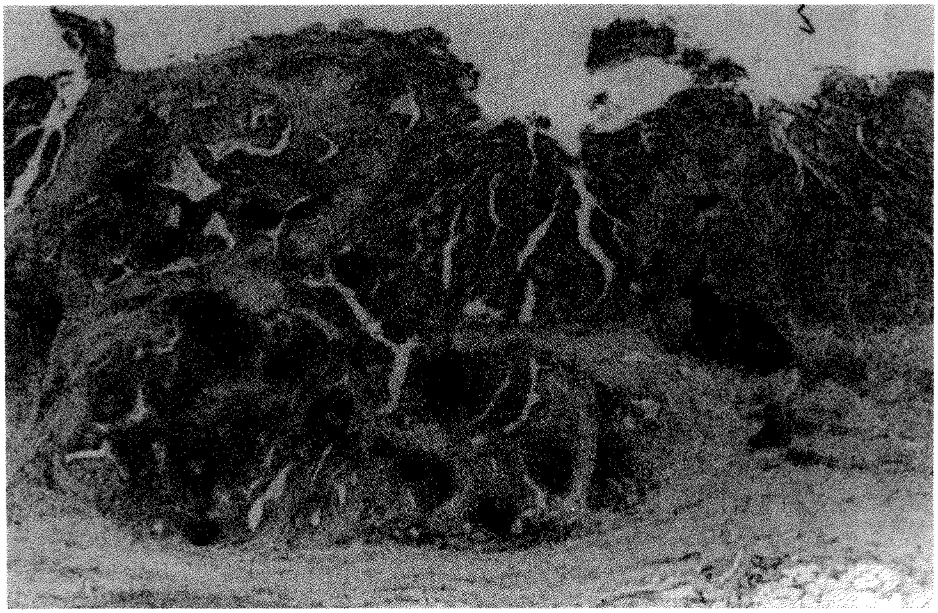

**Figure 2**

**Table 1** The clinical features of 8 patients with cholangitis glandularis proliferans.

| | Patient Number | | | | | | | |
|---|---|---|---|---|---|---|---|---|
| | 1 | 2 | 3 | 4 | 5 | 6 | 7 | 8 |
| Sex | F | M | F | F | F | M | F | M |
| Age (YR) | 44 | 68 | 67 | 44 | 59 | 68 | 31 | 39 |
| Jaundice | + | + | + | + | + | + | + | + |
| Pain | – | – | – | – | – | – | + | – |
| Colitis | – | – | – | – | + | – | – | + |
| Calculi | + | + | + | + | + | + | + | – |
| Carcinoma | – | – | – | – | – | + | – | + |

Certainly, CGP is not uncommon and is thought to result from gall bladder epithelial loss due to altered emptying pressures caused by abnormal contractions associated with an increase in subepithelial mural elements[6–8]. In two of the seven reported cases of CAGP the presence of increased mural tissue was commented upon though this was not seen in our case.

The following clinical information has been recorded in the seven previously reported cases[1,9]. Five of the seven were female with ages ranging from 31 to 68 years. All presented with jaundice which was painless in six of the seven. Operative procedures, on these patients, include resections of part or all of the extrahepatic biliary tree with Roux-en-Y reconstructions, one pancreaticoduodenectomy for benign tumour and one local resection of an adenocarcinoma. This patient died within one year. Interestingly, all seven of these cases also had stones which is distinctly unusual in patients with sclerosing cholangitis. Only one patient had colitis and only one had a co-existent biliary malignancy. The clinical details of all eight cases are summarised in Table 1.

In conclusion, cholangitis glandularis proliferans may be an uncommon variant of sclerosing cholangitis. It does not appear to be related as strongly to inflammatory bowel disease as is fully developed sclerosing cholangitis. This eighth reported case of CAGP, the second such case reported in association with cholangiocarcinoma, highlights a possible significant link between CAGP and biliary malignancy. The possibility of such a link should be kept in mind by clinicians dealing with biliary disease.

### *References*

1. Krukowski, M., McPhie, J.L., Farquharson, A.G.M. and Matheson, N.A. (1983) Proliferative cholangitis (cholangitis glandularis proliferans). *Br. J. Surg.*, **70**, 166–171
2. Aldridge, M.C., Gruffaz, F., Castaing, D. and Bismuth, H. (1991) Adenomyomatosis of the gallbladder. A premalignant lesion? *Surgery.*, Jan, 107–110
3. Spitz, L. and Petropoulos, A. (1979) The development of the glands of the common bile duct. *J. Pathol.*, **128**, 213–220
4. Hoe, T. (1962) Repair of the extrahepatic bile ducts after mechanical and chemical injury. *J. Pathol. Bacteriol.*, **82**, 83–94
5. Eleftheriadis, E., Tzioufa, V., Kotzampassi, K. and Aletras, H. (1988) Common bile-duct mucosa in choledochoduodenostomy patients — histological and histochemical study. *HPB Surgery*, **1**, 15–20
6. Le Quesne, L.P. and Ranger, I. (1957) Cholecystitis glandularis proliferans. *Br. J. Surg.*, **44**, 447–448
7. Woodard, B.H. (1982) Adenomatous hyperplasia of the human gallbladder. *South Med. J.*, **75**, 533–535
8. Jutras, J.A. and Levergue, H.P. (1966) Adenomyoma and adenomyomotosis of the gallbladder. *Radiol. Clin. N. America*, **4**, 483–500
9. Graham, S.M., Barwick, K., Cahow, C.E. and Baker, C.C. (1988) Cholangitis glandularis proliferans. *J. Clin. Gastro.*, **10**(5), 579–583

*(Accepted by S. Bengmark 20 February 1992)*

